# The Pathological Effects of Cardiac Transplantation in a 27-Year-Old Patient Leading to Cardiac Allograft Rejection and End-Stage Renal Disease: A Case Report

**DOI:** 10.7759/cureus.87435

**Published:** 2025-07-07

**Authors:** Zaza Aladashvili, Angela Zavaro, Azary Hernandez, Nadin Abboud, Sergey Gerasim

**Affiliations:** 1 Faculty of Medicine, Tbilisi State Medical University, Tbilisi, GEO; 2 Internal Medicine, Jackson Memorial Hospital, Miami , USA; 3 Medicine, American University of the Caribbean School of Medicine, Miami, USA; 4 Pediatrics, Jackson Memorial Hospital, Miami, USA; 5 Internal Medicine, Jackson Memorial Hospital, Miami, USA; 6 Internal Medicine, American University of the Caribbean School of Medicine, Miami, USA; 7 Critical Care Medicine, Jackson Memorial Hospital, Miami, USA

**Keywords:** cardiac allograft vasculopathy, cardiology, hepatic transplant complications, kidney failure, medical intensive care unit, transplant kidney

## Abstract

Heart transplantation remains the gold standard for patients with end-stage heart failure, offering a median survival of 12 years. However, long-term complications, such as cardiac allograft vasculopathy (CAV), infections, malignancies, and renal dysfunction, pose significant challenges. We present the case of a 27-year-old male who underwent heart transplantation at age 15 for dilated cardiomyopathy and now presents with multisystem failure.

The patient arrived at the emergency department with dyspnea, abdominal distention, and respiratory distress. He experienced pulseless electrical activity and was successfully resuscitated and transferred to the ICU. Echocardiography revealed a reduced ejection fraction (30%-35%), consistent with graft dysfunction. Laboratory results showed severe anemia, elevated blood urea nitrogen (BUN) and creatinine levels, and an estimated glomerular filtration rate (eGFR) of 9 mL/min/1.73 m², consistent with end-stage renal disease (ESRD). He was on hemodialysis and long-term immunosuppressive therapy, including tacrolimus and mycophenolate. Despite stabilization, his prognosis remained poor, prompting evaluation for combined heart and kidney transplantation.

This case highlights the long-term risks of heart transplantation, particularly the development of CAV and ESRD. CAV affects over half of recipients within 10 years and can lead to graft failure, while immunosuppressants contribute significantly to renal decline. Recent data suggest that dual-organ transplantation in patients with severe renal dysfunction improves survival outcomes, particularly among younger candidates. However, such decisions involve ethical considerations around organ allocation and long-term outcomes.

Our patient, due to his age and clinical deterioration, was a strong candidate for dual transplantation. This case underscores the importance of early recognition of post-transplant complications and supports the need for individualized, multidisciplinary approaches. As dual transplants become more common, careful patient selection and continued research are essential to maximize outcomes and ensure ethical distribution of scarce resources.

## Introduction

A heart transplant is a procedure performed in patients with treatment-refractory acute or chronic heart failure [1]. This approach can be the only treatment for patients on inotropic medications, who require mechanical circulation and still have a poor prognosis [1]. Indications for heart transplant in patients with chronic heart failure are readmissions for recurrent heart failure exacerbation despite guideline-directed medical therapy (GDMT), cardio-renal syndrome, left heart failure causing right heart failure and rising pulmonary artery pressure, frequent episodes of ventricular arrhythmias, and other systemic failures caused by heart conditions [1]. Urgent cardiac transplantation indications in acute heart failure include refractory cardiogenic shock despite percutaneous mechanical circulatory support and maximized inotropic therapy, and refractory pulmonary edema requiring mechanical ventilation despite the use of diuretics [1]. Although current statistics show a median survival of 12 years after heart transplantation, and it is considered to offer the best survival benefit for heart failure patients, several complications can still arise from the procedure [2].

Post-transplant complications can be divided into two groups: early and late. Among early complications, primary graft dysfunction is a significant concern. It refers to the failure of graft function within 24 hours of surgery and occurs in approximately 33% of transplant patients [3]. Also, immune-mediated transplant rejection is divided into cell-mediated and antibody-mediated. Although postoperative immunosuppressive therapy has reduced the overall incidence of this complication to 13%, it remains important to manage this adverse event, as it can occur from one year to several decades after surgery [1]. Among delayed complications, cardiac allograft vasculopathy (CAV) is particularly important. It involves the narrowing or occlusion of coronary arteries in the graft and can lead to secondary heart failure [4]. Other important causes are infections and immunosuppressive therapy side effects, regardless of being the single most effective way of maintenance therapy in the post-transplant cohort [1].

One of the interesting clinical problems that transplant patients face is the development of end-stage renal disease (ESRD) in the postoperative course [5]. Following a study from 2020, out of 614 patients who underwent heart transplants, 121 (19.7%) developed ESRD, with a median onset of 7.7 years postoperatively. Among those patients, 80 received dialysis and 19 underwent kidney transplants [5]. The exact reason behind this renal complication is unknown, but several factors can play a role in its development. Risk factors can include a history of hypertension and diabetes, which can progress to long-term inflammatory kidney damage. On the other hand, the immunotherapy used for maintenance in these patients can further damage the kidneys and have adverse effects [5]. Ongoing research at transplant centers is focused on developing dual transplant strategies for patients with heart failure and kidney disease to help prevent future complications [5]. Comorbid ESRD in this patient population poses a significant clinical challenge. As a result, several studies aim to reduce the risk of this complication and improve survival rates in heart transplant recipients.

This case presents a 27-year-old male with a history of heart transplantation who requires dual transplant therapy of the kidney and heart 12 years after surgery.

## Case presentation

A 27-year-old male presented to the emergency department with shortness of breath for one week, along with cough and abdominal distention. On admission, this patient was tachypneic and tachycardic, with a heart rate in the 130s. The patient was found to be in respiratory distress, using accessory muscles for respiration. His past medical history was significant for a cardiac transplant in July 2013 due to dilated cardiomyopathy, complicated by right middle cerebral artery stroke, multiple episodes of cardiac arrest, left ventricular assist device, end-stage kidney disease currently requiring dialysis, chronic anemia, hypothyroidism, and asthma. Current medications on admission included tacrolimus, mycophenolate mofetil, valganciclovir, levothyroxine, and ferrous sulfate.

**Figure 1:**
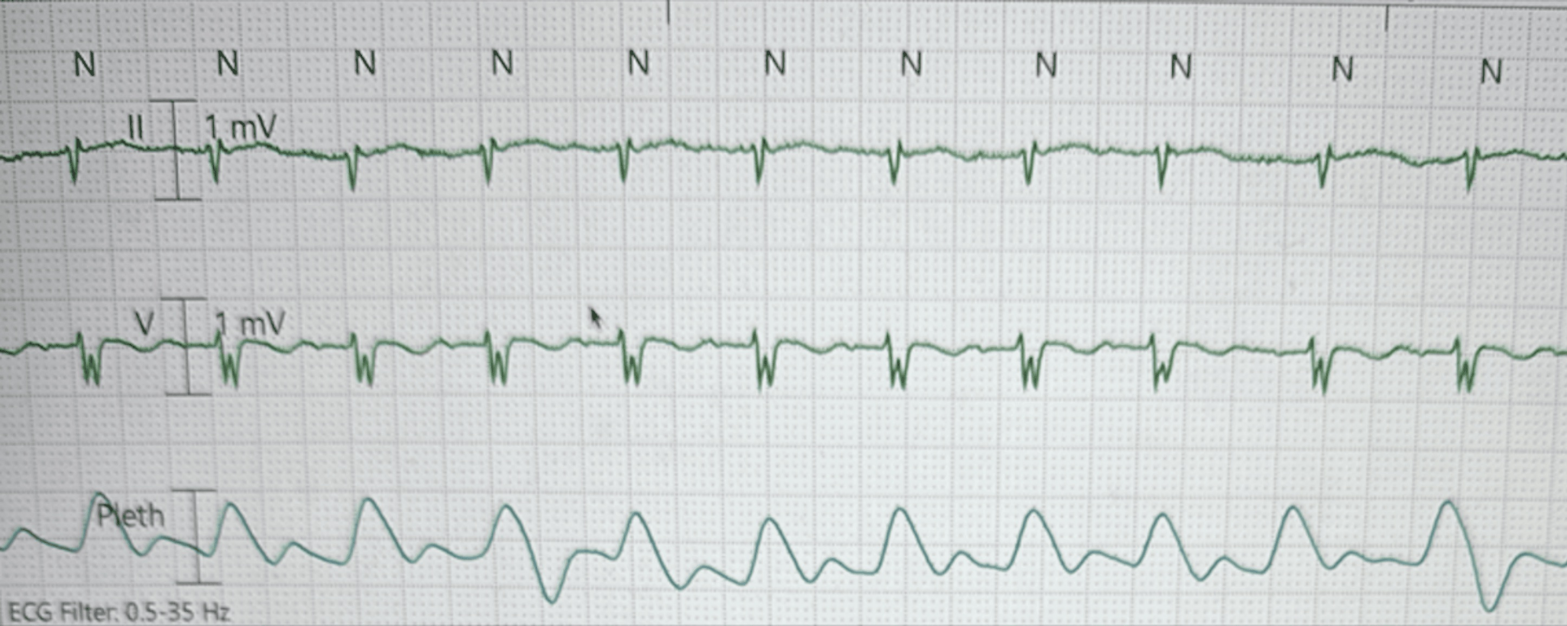
Patient's telemetry strip. Telemetry strip displaying leads II and V, along with the plethysmograph (Pleth) waveform. The strip illustrates cardiac electrical activity alongside peripheral pulse waveforms, aiding in the correlation of heart rhythm with pulse rate.

While under the care of the emergency team, the patient’s condition deteriorated - he became bradycardic, with a heart rate dropping into the 20s, and subsequently developed pulseless electrical activity (PEA). The rapid response team started cardiopulmonary resuscitation (CPR) following advanced cardiac life support (ACLS) guidelines, and resuscitation efforts were successful after six minutes. The patient was stabilized, intubated, placed on a ventilator, and transferred to the intensive care unit (ICU) for further care. The patient’s telemetry strip is shown in Figure 1.

Once transferred to the ICU, cardiac examination noted a grade 3/6 systolic aortic murmur. The patient’s ejection fraction was between 30% and 35%. Abdominal examination revealed a soft abdomen with mild distention. Chest X-ray findings were consistent with pulmonary edema.

This patient’s pertinent labs on admission are illustrated in Table 1.

**Table 1:** Patient's laboratory values during admission. Laboratory results showing the patient’s values compared to the normal reference ranges. Hemoglobin and hematocrit levels are low, indicating anemia. Elevated haptoglobin and fibrinogen suggest an acute-phase response or inflammation. Troponin is mildly elevated, which may indicate cardiac stress or injury. Neutrophil percentage is increased, consistent with possible infection or inflammation. Blood urea nitrogen (BUN), a marker of kidney function and protein metabolism, and creatinine levels are significantly elevated, indicating impaired renal function. Estimated glomerular filtration rate (eGFR), a measure of kidney filtration capacity, is severely decreased. Electrolytes, including sodium, potassium, and phosphorus, are within- or near-normal limits.

Labs	Patient’s value	Reference range
Hemoglobin	7.8 g/dL	Males: 14.0-17.5 g/dL; females: 12.3-15.3 g/dL
Hematocrit	25.3%	Males: 41%-50%; females: 36%-44%
Haptoglobin	408 mg/dL	30-200 mg/dL
Fibrinogen	689 mg/dL	200-400 mg/dL
Troponin	0.085 ng/mL	0-0.04 ng/mL
Neutrophils	84%	40%-60%
BUN	42 mg/dL	6-20 mg/dL
Creatinine	6.30 mg/dL	Males: 0.7-1.3 mg/dL; females: 0.6-1.1 mg/dL
Sodium	135 mEq/L	135-145 mEq/L
Potassium	4.8 mEq/L	3.5-5.2 mEq/L
Phosphorus	4.6 mg/dL	2.5-4.5 mg/dL
eGFR	9 mL/min/1.73 m^2^	≥90 mL/min/1.73 m^2^

The treatment plan for this patient included dobutamine for cardiac inotropic support, heparin for deep vein thrombosis prophylaxis, furosemide (Lasix) for fluid unloading, aspirin, and supportive care to maintain hemodynamic stability. Highlighting this patient’s BUN and creatinine values, dialysis was consistently received and monitored. His immunosuppressive medications played a critical role in his kidney function decline, ultimately leading to organ damage and failure. This led to a series of complications, including anemia. The patient received blood transfusions and iron supplementation, supporting his ongoing effort to achieve stability and possible recovery.

Even though his condition was stabilized by the Medical ICU team, his future prognosis was not promising. The transplant surgery team visited the patient and suggested that the only way for him to start functioning normally would be a heart and renal transplant, as both of these systems showed advanced failure. The patient became a candidate and was placed on the waiting list for organ donation. The complexity of this case highlights the advanced consequences in a heart transplant patient, initially treated for dilated cardiomyopathy, who over time developed multisystem failure as a result of the procedure, ultimately requiring a secondary transplant.

## Discussion

This case involves a 27-year-old male with a history of heart transplantation 12 years ago for dilated cardiomyopathy, who presented with multisystem failure. This included a reduced ejection fraction of 30%-35%, indicating graft dysfunction, heart failure, and pulmonary edema. Additionally, laboratory analysis revealed ESRD, for which the patient was receiving dialysis. His current health condition suggested the need for a repeat transplant, not only of the heart but also a dual transplant, including the kidney.

Complications after transplantation are common and include CAV, malignancies, infections, acute rejection, and renal insufficiency [6]. The pathophysiology of CAV involves progressive vascular stenosis, which leads to graft dysfunction. It develops in one-third of heart transplant patients within five years and in half of them within 10 years. The mortality rate reaches 10% after the third post-transplant year [6]. Despite advances in immunosuppressive therapies, there remains a high susceptibility to CAV. The only way to control its progression is through annual angiographies and the use of statins, which can reduce both CAV-related mortality and disease progression [7]. Malignancies are another common issue in transplant patients, with a 10-year risk of 35% [6]. The most common types are skin cancers, likely as a result of long-term immunosuppressive therapy. On the other hand, infections are the most common early cause of death [6], due to the high doses of immunosuppressive medications used in the early post-transplant period, which leave the immune system vulnerable. As medication doses decrease over time, this risk typically declines. Acute rejection occurs in approximately 10% of patients. Although its incidence is decreasing, it can still contribute to the development of CAV in the future [7]. Finally, drugs such as tacrolimus and cyclosporine are major contributors to the development of ESRD, occurring in 30% of patients within 10 years [6].

In the case of our patient, the most logical cause of his health deterioration was CAV, presenting with fatigue, nausea, and abdominal discomfort. The initial signs of CAV may include a reduced ejection fraction and heart failure or may manifest suddenly as arrhythmia, silent myocardial infarction, or even sudden death, as observed in our patient.

Literature on cardiac transplants and ESRD has become an emerging topic in medical research, as it is not an uncommon complication. In one study conducted between 2006 and 2016, it was estimated that 666 out of 18,982 patients who underwent cardiac transplantation later developed ESRD. Furthermore, the study estimated that the 10-year risk of developing the disease is approximately 9.1% [3]. Patients who develop ESRD post-transplant require hemodialysis multiple times a week for life-sustaining support [8]. The likelihood of receiving a kidney transplant in this context ranges from 0.6% to 3.1% [3]. About 52% of patients are not successful transplant recipients [3]. Several factors influence the possibility of receiving a transplant, leaving many patients dependent on hemodialysis for most of their lives [8]. While dual-organ transplantation is considered, it is based on an individualized approach for each patient. In the patient described in our clinical case report, a dual cardiac and renal transplant will be essential as a lifesaving measure. Additionally, he was a candidate for transplantation, which required strict adherence to his current hemodialysis regimen. Clinical outcomes have improved in recent years to better monitor and prevent early organ damage, particularly ESRD, in post-cardiac transplant patients.

The concept of a heart-kidney transplant is based on the severity of kidney function decline in heart transplant patients. Earlier guidelines suggested that a serum creatinine level greater than 2-3 mg/dL or an eGFR below 40 mL/min/1.73 m² indicated irreversible kidney damage, making heart transplantation alone risky [9]. Modern guidelines recommend a dual transplant in patients with an eGFR below 30 mL/min/1.73 m², or in those with an eGFR between 30 and 44 mL/min/1.73 m² who also show additional signs of chronic kidney disease such as kidney shrinkage, proteinuria, or hyperphosphatemia [9]. Studies show that patients who were candidates for heart transplant with either dialysis dependence or an eGFR below 45 mL/min/1.73 m² had better survival outcomes, with a median of 13 years, compared to those who received a heart transplant alone, whose median survival was 10.2 years [9]. These positive outcomes are most commonly observed in individuals aged 25-45 years [9], suggesting that our patient was a strong candidate, with effective results expected from this management.

This case also highlights important ethical and medical challenges. Allocating two organs (heart and kidney) to a single patient raises questions of fairness, especially when many others are awaiting single-organ transplants. Postoperative risks are heightened in immunosuppressed patients, increasing vulnerability to infections and other complications. The patient’s young age presents a dual reality: on one hand, it offers a longer potential lifespan and greater return on the transplant investment; on the other, it requires lifelong adherence to complex medical regimens and management of chronic side effects. Balancing immediate medical need with long-term societal and ethical considerations is central to evaluating prognosis and transplant candidacy.

This case underscores the important reality that even successful heart transplants do not guarantee permanent stability and, over time, can lead to complications involving multiple organ systems. Early signs of kidney dysfunction and heart failure must be recognized promptly, as delayed action can worsen the prognosis and narrow the window for effective management in this patient population. The case highlights the importance of regular follow-up and a multidisciplinary approach in transplant care. This field still requires further development, as treatment may not lead to complete recovery and can carry long-term complications. However, transplantation remains the only viable treatment option for patients like this, and maximizing its effectiveness is essential.

## Conclusions

This case illustrates the complex and progressive nature of long-term complications following heart transplantation, notably CAV and ESRD secondary to chronic immunosuppression. The patient’s clinical deterioration underscores the importance of vigilant, ongoing surveillance for multisystem involvement in transplant recipients. Dual heart-kidney transplantation represents a viable lifesaving option in selected patients with combined cardiac and renal failure, particularly younger candidates with dialysis dependence. However, the decision to pursue dual transplantation requires careful consideration of ethical, medical, and resource allocation factors. Multidisciplinary management and timely intervention remain critical to improving outcomes and quality of life in this vulnerable population.
